# Beneficial Effects of Canagliflozin in Heart Failure Associated With Cardiac and Surgical Procedures

**DOI:** 10.31083/RCM46719

**Published:** 2026-02-13

**Authors:** Yuliang Dong, Fan Zhou, Liqun Chi, Junsheng Mu

**Affiliations:** ^1^Capital Medical University, 100069 Beijing, China; ^2^Department of Ultrasound, The Third Medical Center of PLA General Hospital, 100039 Beijing, China; ^3^Department of Cardiac Surgery, Beijing Anzhen Hospital, Capital Medical University, Beijing Institute of Heart Lung and Blood Vessel Diseases, 100029 Beijing, China; ^4^Departemnt of Cardiac Surgery, The Third Affiliated Hospital of Xinxiang Medical University, 453000 Xinxiang, Henan, China

**Keywords:** canagliflozin, heart failure, cardiac surgical procedures, sodium-glucose transporter 2 inhibitors

## Abstract

Heart failure is a significant complication following cardiac surgery. While sodium-glucose co-transporter-2 (SGLT2) inhibitors show established benefits in chronic heart failure, their specific role in the perioperative setting remains poorly defined. This review aims to consolidate the current evidence on the beneficial effects and underlying mechanisms of canagliflozin in managing heart failure associated with cardiac surgery. A narrative review of relevant preclinical animal studies and clinical trials was conducted to integrate and summarize the existing data. The evidence demonstrates that canagliflozin confers cardiovascular protection through multifaceted mechanisms, including improved metabolic regulation, favorable hemodynamic effects, and potent anti-inflammatory and anti-fibrotic actions. These mechanisms are highly relevant to mitigating key pathophysiological insults in the perioperative period. While current clinical data are limited to observational studies, they suggest promising benefits for canagliflozin in reducing postoperative cardiovascular complications. Canagliflozin shows considerable potential as a therapeutic agent for patients with heart failure related to cardiac surgery. However, definitive evidence from large-scale, multicenter randomized controlled trials is warranted to confirm its efficacy and safety, and to optimize perioperative management strategies.

## 1. Introduction

Cardiac dysfunction is a common and serious complication following cardiac surgeries such as coronary artery bypass grafting (CABG), significantly increasing patient mortality and the health care burden. Epidemiological data show that approximately 25% of patients develop heart failure (HF) postoperatively, and heart failure with reduced ejection fraction (HFrEF) accounts for a substantial proportion [1]. Risk factors include advanced age, preoperative left ventricular dysfunction (left ventricular ejection fraction [LVEF] <40%), coronary artery disease, diabetes mellitus, chronic kidney disease, and hypertension [1, 2]. The occurrence of heart failure is closely associated with multiple perioperative pathophysiological processes: surgical trauma and cardiopulmonary bypass can trigger a “cytokine storm”, causing myocardial microcirculatory impairment and endothelial dysfunction [3]. Ischemia-reperfusion injury (IR) during surgery leads to the accumulation of reactive oxygen species (ROS), impairing myocardial contractility, exacerbating myocardial remodeling, and promoting cardiomyocyte apoptosis [4, 5].

Current guideline-recommended therapies for heart failure (*2021 ESC Guidelines for the Diagnosis and Treatment of Acute and Chronic Heart Failure*) include angiotensin-converting enzyme inhibitors, beta-blockers, mineralocorticoid receptor antagonists, and sodium-glucose co-transporter-2 (SGLT2) inhibitors [2]. However, traditional drugs have substantial limitations in the postoperative setting, such as intolerance due to hypotension or renal dysfunction, and limited efficacy in regulating metabolic and inflammatory responses [6]. Therefore, novel multi-target therapeutic strategies are urgently needed to overcome these limitations.

SGLT2 inhibitors have emerged as a cornerstone therapy in heart failure. Evidence from both large-scale clinical trials [7, 8] and multiple meta-analyses [9, 10, 11] confirms their ability to significantly reduce cardiovascular death, myocardial infarction, heart failure events, and all-cause mortality in high-risk patients, providing robust evidence for their cardioprotective effects. As one of the earliest SGLT2 inhibitors, canagliflozin has proven benefits in patients with type 2 diabetes and high cardiovascular risk.

However, the robust evidence for SGLT2 inhibitors is primarily derived from studies in stable, non-surgical populations. The unique pathophysiological context of cardiac surgery—marked by profound inflammation, ischemia-reperfusion injury, and hemodynamic instability—presents a critical knowledge gap regarding their perioperative efficacy and safety. This article provides a narrative review of the topic, based on a literature search of the PubMed and Embase databases for relevant studies. It aims to consolidate the current preclinical and clinical evidence for canagliflozin, summarizing its multifaceted mechanisms and potential role in managing heart failure associated with cardiac surgery.

## 2. Beneficial Effects and Potential Mechanisms of Canagliflozin

**Fig. 1.**
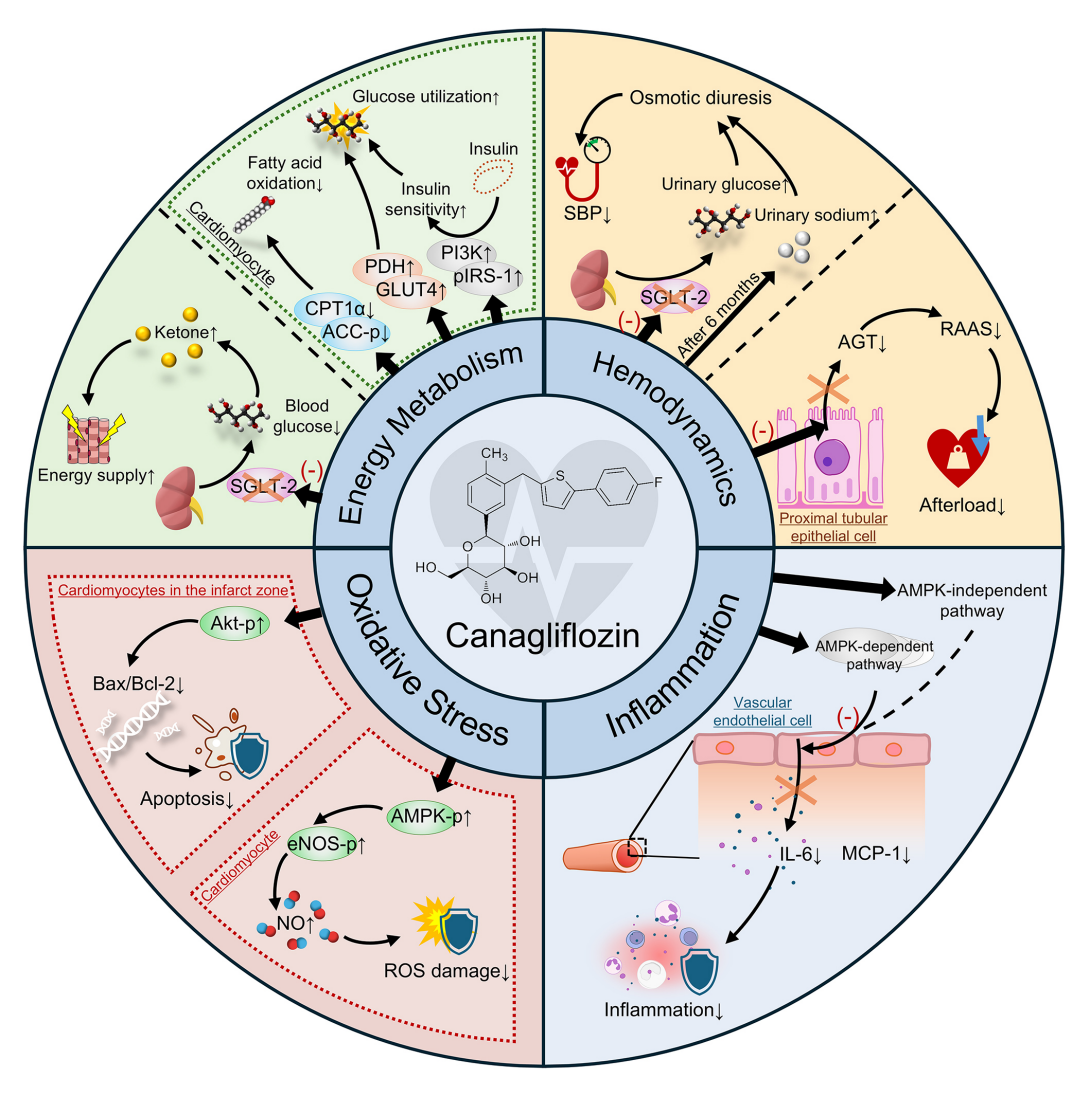
**Mechanisms of canagliflozin in improving cardiac function**. SGLT-2, Sodium-Glucose Co-Transporter 2; ACC-p, phosphorylated acetyl CoA carboxylase; CPT1α, carnitine palmitoyltransferase 1 alpha; PDH, pyruvate dehydrogenase complex; GLUT4, glucose transporter type 4; PI3K, phosphoinositide 3-kinase; pIRS-1, phosphorylated insulin receptor substrate-1; SBP, systolic blood pressure; AGT, angiotensinogen; RAAS, renin-angiotensin-aldosterone system; AMPK, AMP-activated protein kinase; MCP-1, monocyte chemoattractant protein-1; IL-6, interleukin-6; ROS, reactive oxygen species; NO, nitric oxide; eNOS, endothelial nitric oxide synthase; Bax, BCL2 associated X protein; Bcl-2, B-cell lymphoma-2 gene; Akt, protein kinase B. “↑⁣/⁣↓” stand for “increase/decrease”.

As an SGLT2 inhibitor, canagliflozin improves cardiac function via mechanisms beyond glycemic control, including metabolic regulation and hemodynamic modulation as well as anti-inflammatory and antifibrotic effects, resulting in comprehensive cardiovascular protection (Fig. 1, Table 1).

**Table 1.** **Summary of beneficial mechanisms of canagliflozin relevant to post-cardiac surgery patients**.

Category	Specific mechanism	Relevance to post-cardiac surgery patients
Hemodynamic	Reduces cardiac preload and blood pressure through osmotic diuresis and natriuresis.	Alleviates common postoperative fluid overload and congestion, reducing stress on the recovering heart.
	Lowers vascular resistance and afterload by downregulating the RAAS.	Decreases the workload on the surgically stressed heart, improving cardiac efficiency and reducing oxygen demand.
Metabolic regulation	Induces the production of ketone bodies, a highly efficient energy substrate for cardiomyocytes.	The heart is under significant metabolic stress post-surgery. Ketones act as a “super fuel” that can improve cardiac efficiency and function during this critical recovery period.
	Promotes a shift from fatty acid oxidation to more efficient glucose utilization and enhances insulin signaling.	Counters maladaptive metabolic responses in the stressed myocardium, improving overall energy homeostasis.
Anti-inflammatory effects	Reduces the release of pro-inflammatory cytokines such as IL-6 and MCP-1.	Mitigates the systemic “cytokine storm” often triggered by cardiopulmonary bypass and surgical trauma.
	Activates the* Nrf2* antioxidant pathway to mitigate cellular damage from ROS.	Protects cardiomyocytes from ischemia-reperfusion injury, a key pathological event during cardiac surgery.
Direct myocardial & Cellular effects	Exerts anti-fibrotic effects by reducing myocardial collagen deposition and cardiac stiffness.	Helps prevent adverse cardiac remodeling following surgical injury, improving long-term diastolic function.
	Reduces cardiomyocyte apoptosis by activating pro-survival signaling pathways like Akt.	Preserves viable myocardial tissue at risk from the intense stress of the perioperative period.

RAAS, Renin-Angiotensin-Aldosterone System; IL-6, interleukin-6; MCP-1, monocyte chemoattractant protein-1; *Nrf2*, nuclear factor erythroid 2; ROS, reactive oxygen species; Akt, protein kinase B.

Canagliflozin inhibits SGLT2 in renal tubules, promoting glycosuria, indirectly reducing blood glucose levels, and inducing ketone body production. Ketone bodies serve as alternative energy substrates for cardiomyocytes, providing an efficient source of adenosine triphosphate under ischemic or energy-deficient conditions, thereby enhancing myocardial energy metabolism. Clinical studies have shown that plasma ketone levels increase and cardiac function improves after canagliflozin treatment in patients with mild cardiac dysfunction [12]. In a porcine model of myocardial ischemia, canagliflozin suppressed myocardial fatty acid oxidation and promoted glucose utilization, enhanced insulin signaling in ischemic myocardium, reduced insulin resistance, and improved myocardial lipid metabolism [13].

Through osmotic diuresis and natriuresis, canagliflozin effectively lowers blood pressure and reduces cardiac preload [14]. It also suppresses renal angiotensinogen production by inhibiting SGLT2 activity, thereby downregulating the renin–angiotensin–aldosterone system and reducing vascular resistance and afterload [15, 16].

Canagliflozin inhibits the release of pro-inflammatory cytokines such as interleukin-6 (IL-6) and monocyte chemoattractant protein-1 (MCP-1) in endothelial cells via pathways that are both dependent and independent of adenosine monophosphate-activated protein kinase, significantly reducing myocardial and vascular inflammation [17, 18]. Furthermore, canagliflozin activates the nuclear factor erythroid 2 (*Nrf2*)-related factor 2 pathway, enhances antioxidant enzyme activities (e.g., superoxide dismutase, glutathione peroxidase), and mitigates ROS-induced myocardial oxidative damage [19]. In a rat myocardial infarction model, pretreatment with canagliflozin activated the adenosine monophosphate-activated protein kinase and protein kinase B (Akt) pathways, promoted nitric oxide production, reduced cardiomyocyte apoptosis, and significantly reduced infarct size [20].

## 3. Effects of Canagliflozin on Cardiac Surgery-Associated Heart Failure

### 3.1 Animal Experimental Evidence

#### 3.1.1 Metabolic Reprogramming

In a porcine model of chronic myocardial ischemia, canagliflozin improved cardiac function by regulating myocardial metabolism. After 5 weeks of treatment, key enzymes in fatty acid oxidation such as carnitine palmitoyltransferase 1α and phosphorylated acetyl-coenzyme A (acetyl-CoA) carboxylase were significantly downregulated (*p* < 0.05), and the expression of pyruvate dehydrogenase complex and glucose transporter 4 was upregulated (*p* < 0.05), indicating suppression of fatty acid oxidation and promotion of glucose utilization [13]. Canagliflozin also enhanced the insulin signaling pathway (elevated phosphatidylinositol 3-kinase and phosphorylated insulin receptor substrate-1 expression), thereby reducing myocardial insulin resistance and offering a therapeutic target for postoperative energy metabolism disorders [13].

This finding is strongly supported by a recent study in a large animal model. Stone *et al*. [21] used an ischemic swine model and found that SGLT2 inhibitor treatment for 5 weeks led to a nearly 1.5-fold increase in both cardiac output and ejection fraction. These functional improvements were associated with a twofold increase in perfusion to the ischemic myocardium and were linked to the modulated expression of multiple key metabolic enzymes, confirming the crucial role of metabolic normalization in the ischemic heart [21].

#### 3.1.2 Anti-Inflammatory and Antioxidative Effects

In a non-diabetic Lewis rat model of aortic bypass surgery with IR injury, canagliflozin significantly protected vascular graft function by preserving endothelial integrity. IR injury reduced endothelium-dependent vasodilation to 20% (vs. 83% in controls, *p* < 0.05). Perioperative intravenous injection of canagliflozin (10 µg/kg) or supplementation in graft preservation fluid (5 µM) partially restored endothelium-dependent vasodilation (36%–45%), with combined application showing improved efficacy (*p* < 0.05) [22]. Immunohistochemistry revealed reduced IR-induced deoxyribonucleic acid (DNA) damage and increased expression of the endothelial marker cluster of differentiation 31 (CD-31), indicating antioxidative and endothelial-protective effects [22].

In an *ApoE⁻/⁻* murine model of atherosclerosis, canagliflozin significantly inhibited inflammation and oxidative stress. Combined with a high-fat diet, this agent reduced serum pro-inflammatory cytokines (tumor necrosis factor α, IL-6, IL-1β) (*p* < 0.05) and macrophage infiltration (F4/80+ cells) in plaques (*p* < 0.05) [18]. Mechanistically, canagliflozin upregulated antioxidant genes such as *Nrf2* and endothelial nitric oxide synthase, reducing oxidative stress markers including ROS and nicotinamide adenine dinucleotide phosphate oxidase 4, thereby enhancing antioxidative defenses [18].

#### 3.1.3 Antifibrotic Effects

In a rat model of heart failure with preserved ejection fraction (HFpEF), canagliflozin improved diastolic function by modulating ferroptosis pathways. Rats with HFpEF induced by eating a high-salt diet, exhibited increased myocardial iron deposition (Prussian blue staining) and the lipid peroxidation marker, malondialdehyde (*p* < 0.05). Canagliflozin downregulated the ferroptosis-related proteins transferrin receptor 1 and acyl-CoA synthetase long chain family member 4, and upregulated ferritin heavy chain, thereby mitigating mitochondrial damage [23]. Masson staining showed reduced myocardial collagen volume fraction (*p* < 0.05), indicating reduced myocardial stiffness via antifibrotic mechanisms [23].

### 3.2 Clinical Research Progress 

#### 3.2.1 Inflammation Inhibition and Cardiovascular Outcomes

The clinical evidence supporting the use of SGLT2 inhibitors in the cardiac surgery setting, while still emerging, is promising. Early cohort studies provided initial insights. A multicenter cohort study by Sardu *et al*. [24] among patients with type 2 diabetes (T2DM) undergoing minimally invasive extracorporeal circulation (MiECC) CABG showed that long-term SGLT2 inhibitor use (≥6 months) significantly lowered postoperative inflammatory markers (IL-6, tumor necrosis factor α) (*p* < 0.05) and improved 5-year outcomes. Compared with non-users of SGLT2 inhibitors, the incidence of composite endpoints (all-cause death, heart failure hospitalization) decreased by 50% (hazard ratio [HR] = 0.504, 95% confidence interval [CI] 0.078–0.861), with systemic inflammation and oxidative stress inhibition contributing to delayed ventricular remodeling [24].

A study by Liu *et al*. [25] involving 224 elderly patients with type 2 diabetes after bioprosthetic valve replacement found that canagliflozin reduced the 1-year risk of major adverse cardiovascular events (MACE) by 21% (HR = 0.792, 95% CI 0.568–0.959, *p* = 0.028), including a 19% reduction in hospitalization for heart failure (HR = 0.807, *p* = 0.033).

While these studies focused on long-term outcomes, more direct evidence on the acute postoperative phase comes from a recent pilot study by Labaste *et al*. [26]. In a propensity score-matched cohort of patients with reduced ejection fraction undergoing cardiac surgery, preoperative SGLT2 inhibitor use was associated with a significantly lower incidence of postoperative cardiovascular complications compared to controls (odds ratio [OR] =0.51, *p* = 0.04). This effect was primarily driven by a substantial reduction in postoperative myocardial injury (PMI) (OR = 0.46, *p* = 0.04) [26]. This finding is particularly important as it provides direct evidence of cardioprotection in the immediate perioperative period.

#### 3.2.2 Cardiac Function Improvement

The Canagliflozin: Impact on Health Status, Functional Status and Quality of Life in Heart Failure (CHIEF-HF) trial enrolled 476 patients with heart failure (28% with diabetes) and demonstrated that canagliflozin significantly improved symptom scores (mean Kansas City Cardiomyopathy Questionnaire [KCCQ] Total Symptom Score increase of 4.3, 95% CI 0.8–7.8, *p* = 0.016) within 12 weeks. These effects were consistent in patients who had heart failure with reduced ejection fraction as well as those with HFpEF [8]. Although not limited to surgical populations, these findings indirectly support the use of canagliflozin in post-cardiac surgery heart failure.

Another single-center study of 80 patients with chronic heart failure showed that 12-week adjunctive treatment with canagliflozin significantly improved left ventricular ejection fraction (*p* < 0.05) and reduced myocardial energy expenditure as well as levels of N-terminal pro B-type natriuretic peptide (*p* < 0.05), suggesting benefits for cardiac energy metabolism [27].

#### 3.2.3 Synergy With Conventional Medications

Jain *et al*. [28] conducted a pooled analysis of the CANVAS and CREDENCE trials and confirmed that canagliflozin further reduced the risks of heart failure hospitalization and cardiovascular death in patients already receiving beta-blockers and renin–angiotensin–aldosterone system inhibitors (RAASi) (HR = 0.86, 95% CI 0.75–0.97), regardless of diabetes or renal status. This supports the role of canagliflozin in combination therapy for post-cardiac surgery management [28].

#### 3.2.4 Synthesis of Clinical Evidence

In critically analyzing the available clinical data, a distinction must be made. The direct evidence in cardiac surgery patients (e.g., Sardu *et al*. [24], Liu *et al*. [25], and Labaste *et al*. [26]) is derived from observational or retrospective cohort studies, which, while valuable, are susceptible to inherent biases. Conversely, high-quality evidence from large-scale randomized controlled trials such as CHIEF-HF and the CANVAS program robustly supports canagliflozin’s benefits, but in non-surgical or chronic heart failure populations. Therefore, while the collective evidence consistently suggests a cardioprotective potential in the perioperative setting, this conclusion relies on integrating indirect high-level evidence with direct but methodologically limited studies (Table 2, Ref. [7, 8, 24, 25, 26, 27, 28]).

**Table 2.** **Summary of clinical studies**.

Author	Publication year	Patient number	Objective	Conclusion
Sardu *et al*. [24]	2021	648	To evaluate the effects of SGLT2 inhibitors on inflammatory burden and 5-year clinical outcomes in MiECC CABG patients.	Long-term SGLT2 inhibitor use reduced 5-year composite endpoint events (death/HF hospitalization) and postoperative inflammation.
Liu Yingying *et al*. [25]	2022	224	To assess the impact of canagliflozin on 1-year cardiovascular outcomes in elderly T2DM patients after valve replacement.	Reduced 1-year MACE risk in elderly T2DM patients after bioprosthetic valve replacement.
Labaste *et al*. [26]	2025	172	To describe the impact of SGLT2i on cardiovascular outcomes in patients undergoing cardiac surgery.	Preoperative SGLT2i use was associated with fewer postoperative cardiovascular complications, driven by reduced myocardial injury.
Spertus *et al*. [8]	2022	476	To evaluate the effects of canagliflozin on health status (symptoms) in various heart failure patients.	Significantly improved heart failure symptom scores (KCCQ) at 12 weeks in patients with both HFrEF and HFpEF.
Zhou Yanhua *et al*. [27]	2024	80	To investigate the regulatory effects of canagliflozin on myocardial energy metabolism and its clinical efficacy in heart failure patients.	Improved LVEF and myocardial energy metabolism at 12 weeks in chronic heart failure patients on conventional therapy.
Jain *et al*. [28]	2024	14,543	To assess the therapeutic effects of canagliflozin in heart failure patients already receiving standard therapy.	Further reduced HF hospitalization and CV death in patients already on standard RAASi and beta-blocker therapy.
Neal *et al*. [7]	2017	10,142	To evaluate the effects of canagliflozin on cardiovascular and renal events, as well as safety.	Reduced major composite endpoint events and slowed kidney disease progression in high-risk T2DM patients; noted an increased amputation risk.

SGLT2, sodium-glucose co-transporter-2; T2DM, type 2 diabetes; SGLT2i, sodium-glucose co-transporter-2 inhibitor; MACE, major adverse cardiovascular events; HFrEF, Heart Failure with Reduced Ejection Fraction; HFpEF, heart failure with preserved ejection fraction; CV, cardiovascular; RAASi, renin–angiotensin–aldosterone system inhibitors; MiECC, minimally invasive extracorporeal circulation; CABG, coronary artery bypass grafting; LVEF, left ventricular ejection fraction.

### 3.3 Drug Safety

The safety profile of canagliflozin in patients undergoing cardiac surgery warrants particular attention regarding the risks of diabetic ketoacidosis (DKA) and infections, as well as strategies for perioperative drug management to balance risks and benefits.

Several studies indicate that canagliflozin increases the risk of euglycemic DKA following cardiac surgery. A retrospective analysis by Brekke *et al*. [29] found that patients in the canagliflozin group exhibited significantly lower base excess 12 hours postoperatively, suggesting an increased risk of ketone production, especially in those with normal renal function. A recent meta-analysis further confirmed that canagliflozin presents a higher DKA risk compared with other SGLT2 inhibitors (odds ratio = 1.11, 95% CI 1.11–12.45), particularly in patients who have had diabetes longer than 10 years [30]. Infection risk is also distinct with canagliflozin, particularly urinary tract infections (UTIs) and mycotic genital infections (MGIs). Research shows that canagliflozin increases the risk of serious UTIs by 13% compared with empagliflozin (HR = 1.13, 95% CI 1.03–1.24), with women being more susceptible to MGIs and UTIs than men. This may be related to partial SGLT1 inhibition and alterations in the gut microbiome [31, 32]. World Health Organization pharmacovigilance data indicate a higher reporting rate of specific infections such as osteomyelitis and cellulitis among canagliflozin users compared with users of other SGLT2 inhibitors, underscoring the need for vigilant postoperative infection monitoring and prompt antimicrobial intervention, if necessary [33].

Although the CANVAS trial reported a potential increase in amputation risk (HR = 1.97), this was mainly observed in patients with diabetes and peripheral arterial disease [7]. In contrast, Liu *et al*. [25] found no significant difference in adverse event rates between the canagliflozin and control groups, suggesting that with careful patient selection and perioperative management, canagliflozin can be used with acceptable safety in surgical settings.

Preoperative discontinuation strategies are crucial. Current guidelines recommend stopping canagliflozin 3 days before surgery to minimize the risks of hypovolemia and DKA [34]. However, Auerbach *et al*. [35] demonstrated that a 5-day discontinuation period could effectively prevent postoperative euglycemic DKA, without increasing infection or mortality risks. Therefore, for high-risk individuals (e.g., those with long-standing diabetes, metabolic instability, or planned complex cardiac procedures), extending the discontinuation to 5 days is advisable. Postoperative reinitiation should be based on a comprehensive evaluation of hemodynamic stability and acid–base balance.

Perioperative metabolic monitoring should cover the preoperative, intraoperative, and postoperative phases [29]. Particular attention should be paid to the electrolyte and acid–base balance, especially in patients with preserved renal function who are at greater risk of ketosis. Increased monitoring frequency is recommended for patients with incomplete preoperative drug withdrawal or intraoperative hemodynamic fluctuations. Although drug discontinuation may temporarily increase the risk of postoperative hyperglycemia, this does not translate into worsened clinical outcomes [35] and may be mitigated through intensive glycemic management.

Identifying high-risk patients is key to optimizing safety. Apart from long-standing diabetes (>10 years) and preserved renal function, patients with a history of DKA, recurrent UTIs or MGIs, peripheral arterial disease, and female patients require special precautions [7, 32, 33]. Multidisciplinary assessment is recommended preoperatively to establish individualized drug regimens, and postoperative education that empowers patients to recognize the early signs of infection (e.g., dysuria, pruritus) should be provided for timely intervention.

## 4. Limitations

This review has several limitations that should be acknowledged. First and foremost, this is a narrative review, not a systematic review or meta-analysis. The selection of studies was based on the authors’ expertise and their relevance to the topic, rather than a predefined, exhaustive search protocol such as PRISMA. This approach is susceptible to potential selection bias, and the synthesis of evidence is qualitative rather than quantitative.

Second, the primary clinical evidence directly addressing the use of canagliflozin in the cardiac surgery setting is still limited and primarily consists of small-scale, observational, or retrospective cohort studies. Such study designs are susceptible to inherent confounding biases, even when statistical adjustments like propensity score matching are used. Consequently, the strength of the evidence is not yet sufficient to draw definitive conclusions regarding hard clinical endpoints such as mortality.

Third, a significant limitation is the generalizability of findings from large-scale randomized controlled trials in non-surgical populations to patients undergoing cardiac surgery. Major trials like CANVAS and CHIEF-HF enrolled patients with chronic conditions, whose stable pathophysiological state differs markedly from the acute, high-stress milieu of the perioperative period, which is characterized by IR injury, systemic inflammation from cardiopulmonary bypass, and profound hemodynamic fluctuations. Extrapolating the benefits observed in chronic settings to the acute surgical context must be done with caution.

Finally, this review discusses “cardiac surgery” as a broad category, which overlooks the significant heterogeneity among different procedures (e.g., CABG vs. valve replacement) and patient subgroups (e.g., diabetic vs. non-diabetic, HFrEF vs. HFpEF). The effects of canagliflozin may differ across these varied clinical scenarios, and the current literature is insufficient to provide tailored recommendations for each specific group.

## 5. Challenges and Future Perspectives

Several challenges must be addressed before canagliflozin can be widely adopted in the perioperative setting. Although its mechanisms—targeting energy metabolism, hemodynamics, inflammation, and fibrosis—offer a comprehensive approach to managing cardiac dysfunction post-surgery, these mechanisms are not fully elucidated. Further investigation is required to verify their roles through experimental and clinical studies. Additionally, optimal timing and dosing strategies for the perioperative use of canagliflozin remain undefined. Whereas preoperative discontinuation clearly reduces DKA risk, the ideal timing for postoperative reinitiation requires further exploration.

Future efforts should include large-scale, multicenter randomized controlled trials involving diverse cardiac surgery procedures (e.g., CABG, valve replacement) and broader populations (including patients without diabetes). Such trials should also extend the follow-up duration to comprehensively evaluate the long-term cardiovascular efficacy and safety of canagliflozin. In future research, the heterogeneity of surgical patients (e.g., procedure type, baseline cardiac function, comorbidities) should be considered so as to define the therapeutic boundary. Detailed data collection and mechanistic studies are needed to clarify the multi-target effects of canagliflozin and inform personalized perioperative management strategies, including preoperative withdrawal, postoperative reinitiation, and combination with conventional therapies.

## 6. Conclusion

In conclusion, canagliflozin presents a compelling therapeutic potential for managing heart failure associated with cardiac surgery. Its beneficial effects are supported by a strong mechanistic rationale spanning hemodynamic, metabolic, and anti-inflammatory pathways, and are corroborated by promising evidence from preclinical and early clinical studies. However, the current clinical evidence is not yet definitive and is primarily derived from non-randomized studies. While balancing its benefits against safety risks such as DKA remains a key consideration, canagliflozin stands out as a promising agent. Future large-scale randomized controlled trials are essential to firmly establish its role and guide its integration into perioperative care for this high-risk patient population.

## Abbreviations

ACEI, Angiotensin-Converting Enzyme Inhibitor; AGT, Angiotensinogen; AMPK, AMP-Activated Protein Kinase; CABG, Coronary Artery Bypass Grafting; CANVAS, Canagliflozin Cardiovascular Assessment Study; CHIEF-HF, Canagliflozin: Impact on Health Status, Functional Status and Quality of Life in Heart Failure; CPT1α, Carnitine Palmitoyltransferase 1 Alpha; DKA, Diabetic Ketoacidosis; eDKA, Euglycemic Diabetic Ketoacidosis; eNOS, Endothelial Nitric Oxide Synthase; ESC, European Society of Cardiology; FTH1, Ferritin Heavy Chain 1; GLUT4, Glucose Transporter Type 4; HFpEF, Heart Failure with Preserved Ejection Fraction; HFrEF, Heart Failure with Reduced Ejection Fraction; IL-1β, Interleukin-1 Beta; IL-6, Interleukin-6; IR, Ischemia-Reperfusion; KCCQ-TSS, Kansas City Cardiomyopathy Questionnaire-Total Symptom Score; LVEF, Left Ventricular Ejection Fraction; MACE, Major Adverse Cardiovascular Events; MEE, Myocardial Energy Expenditure; MGIs, Mycotic Genital Infections; MiECC, Minimally Invasive Extracorporeal Circulation; MRAs, Mineralocorticoid Receptor Antagonists; NADPH, Nicotinamide Adenine Dinucleotide Phosphate; NO, Nitric Oxide; NOX4, NADPH Oxidase 4; NT-proBNP, N-terminal pro-B-type Natriuretic Peptide; OR, Odds Ratio; pACC, Phosphorylated Acetyl-CoA Carboxylase; PDH, Pyruvate Dehydrogenase Complex; PI3K, Phosphoinositide 3-Kinase; pIRS-1, Phosphorylated Insulin Receptor Substrate-1; RAAS, Renin-Angiotensin-Aldosterone System; ROS, Reactive Oxygen Species; SGLT2, Sodium-Glucose Co-Transporter 2; T2DM, Type 2 Diabetes Mellitus; TNF-α, Tumor Necrosis Factor Alpha; TFR1, Transferrin Receptor 1; UTIs, Urinary Tract Infections; CD-31, cluster of differentiation 31; DNA, deoxyribonucleic acid; CV, cardiovascular.
